# Garcinia mangostana extract and curcumin ameliorate oxidative stress, dyslipidemia, and hyperglycemia in high fat diet-induced obese Wistar albino rats

**DOI:** 10.1038/s41598-021-86545-z

**Published:** 2021-03-31

**Authors:** Ranyah Shaker M. Labban, Hanan A. Alfawaz, Ahmed T. Almnaizel, May N. Al-Muammar, Ramesa Shafi Bhat, Afaf El-Ansary

**Affiliations:** 1 Department of Food Science and Nutrition, College of Food and Agriculture Sciences, King Saud University, Riyadh, Saudi Arabia; 2 Ministry of Health, General Administration of Nutrition, Riyadh, Saudi Arabia; 3 Prince Naif for Health Research Center, King Saud University, Riyadh, Saudi Arabia; 4 Department of Community Health, College of Applied Medical Sciences, King Saud University, Riyadh, Saudi Arabia; 5 Biochemistry Department, College of Science, King Saud University, Riyadh, Saudi Arabia; 6 Central Laboratory, Female Centre for Scientific and Medical Studies, King Saud University, Riyadh, Saudi Arabia

**Keywords:** Biochemistry, Drug discovery

## Abstract

The aim of this study was to explore the effects of *Garcinia mangostana* (mangosteen) and *Curcuma longa* independently and synergistically in modulating oxidative stress, dyslipidemia, and hyperglycemia commonly observed in high-fat diet-induced obesity in rodent models. Male albino Wistar rats were divided into eight experimental groups, fed on a normal diet or high-fat diet (HFD), then given mangosteen extract (400 mg /kg /day) and/or curcumin (80 mg/kg /day) for 6 weeks. Oxidative stress markers, glucose, and lipid fractions were measured in the sera. Mangosteen pericarp extract (MPE) induced a remarkable decrease in BMI (from 0.86 to 0.81 gm/cm^2^), while curcuma either alone or in combination was more effective, as treated rats recorded BMIs of 0.78 and 0.79 gm/cm^2^, respectively. Regarding the antioxidant effects, MPE induced a significant increase of GSH in obese rats (123.86 ± 15.53 μg/ml vs 288.72 ± 121.37 μg/ml). As anti-atherogenic agents MPE demonstrate significant effect recorded higher level of HDL-C in treated animals, but ineefective as anti-dyslipidemic agent. Curcumin was more effective in reducing LDL-C levels in obese rats. Both extracts effectively reduced blood glucose. The present study demonstrated that MPE and curcumin were independently and synergistically effective in treating obesity-induced atherogenesis.

## Introduction

Obesity is a complex metabolic disorder with multifactorial etiology. Obesity can lead to reduced quality of life due to numerous associated complications, including cardiovascular disease, diabetes, cancer, asthma, sleep disorders, liver and renal dysfunction, and sterility. Increasing evidence suggests that oxidative stress plays a critical role in linking obesity to these associated complications^1–3^.

Recent studies have proposed that the causes of oxidative stress in obesity include hyperleptinemia, hyperglycemia, insufficient antioxidant capacity, increased free radical generation, mitochondrial dysfunction, and chronic inflammation^4,5^. Accumulating evidence also shows that the quality of diet has demonstrated a negative shift toward consumption of high-energy foods, while intake of nutrient-rich foods such as fruits and vegetables has decreased^6,7^. Such low-quality diets have been linked to a higher risk of obesity^8^ consequently, dietary modification is frequently recommended as the main approach in prevention and treatment of obesity^9,10^. Many studies have also indicated that natural products such as phytochemicals in fruits and vegetables can be vital modulators of the risks accompanying obesity.

Mangosteen (*Garcinia mangostana L.*) fruit has a unique sweet–sour taste, and is rich in valuable compounds, such as xanthones. Mangosteen has been used in various traditional remedies to treat fever, diarrhea, and to promote wound healing. Recently, it has been used as a chief constituent in health supplements for weight loss and for supporting overall health^11^, due to its well-known anti-oxidative and anti-inglammatory medicinal properties^12–14^. Mangosteen pericarp contains mostly sugars (nearly 50% of total metabolites), followed by additional metabolites such as organic acids, alcohols, sugar acids, and aromatic compounds. Additionally, mangosteen pericarp contains several phenolic compounds, such as benzoic acid, tyrosol, and protocatechuic acid, which are known to have anti-oxidative and anti-inflammatory effects.

Interestingly, various studies have revealed that spices such as curcumin may show efficacy in controlling weight gain or reducing obesity. Curcumin is a bioactive polyphenol component found in turmeric rhizomes^15^. Clinical effects of curcumin in reducing weight and body mass index (BMI) have not been thoroughly assessed, and the results of many studies are unreliable and contradictory. Multiple studies have reported that a bioavailable form of curcumin resulted in improved weight management in obese humans and experimental animals^16,17^.

High-energy diets have been utilized to induce obesity and related metabolic disorders in rodent models; however, dietary mediation has not been absolutely standardized^18^. Usually, these diets comprise a simple exchange of carbohydrate-derived calories with fat-derived calories and are compared with a standard chow diet (SCD) as a control.

In this study, we sought to determine the potency of *Garcinia mangostana* pericarp extract and curcumin either independently or synergistically in modulating biochemical markers of oxidative stress, hyperlipidemia, and hyperglycemia in a high-fat diet-induced rodent model of obesity.

## Results

### Effect of treatments on weight gain and BMI

**Figure 1:**
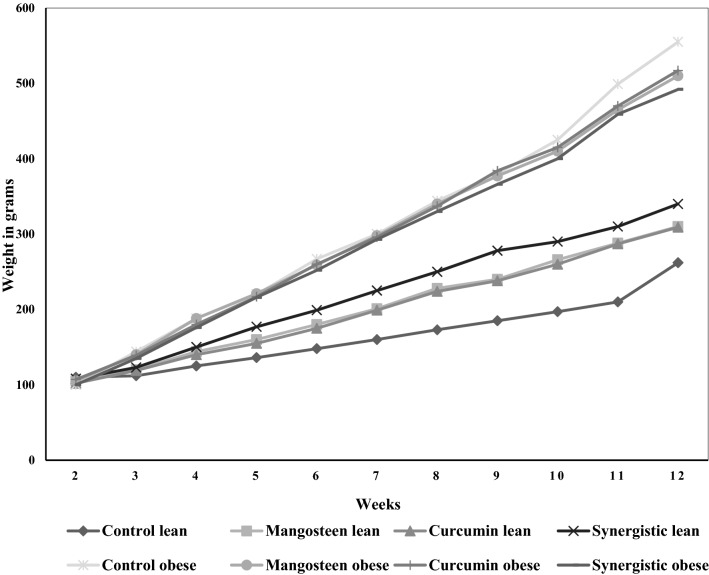
Body weight gain in different groups.

Body weight, weight gain, and BMI in normal and HFD-induced obese rats in response to mangosteen extract and curcumin are shown in Table 1 and Fig. 1. There was a significant difference between normal and obese experimental groups in weight (*p* ˂ 0.001). While the BMI in obese controls (Group 5) reached 0.86 ± 0.09 gm/cm^2^, the normal diet controls (Group 1) recorded a significantly lower BMI of 0.55 ± 0.04 gm/cm^2^ (*p* ˂ 0.001). Both mangosteen extract and curcumin ameliorated the increase in BMI as measure of obesity; while MPE induced a remarkable decrease in BMI (from 0.86 to 0.81 gm/cm^2^), curcuma either alone or in combination was more effective, as BMIs were reduced to 0.78 and 0.79 gm/cm^2^, respectively.

**Table 1:** Independent and synergistic effects of curcumin and mangosteen extracts on BMI of lean and HFD-induced obese rats.

Parameters	Groups	Mean ± SD	p-value^a^	p-value^b^
BW (g)	Control lean	306.80 ± 32.38		
	Mangosteen lean	341.00 ± 70.84		0.355
	Curcumin lean	360.00 ± 52.56		0.090
	Synergistic lean	317.00 ± 15.67		0.544
	Control obese	528.40 ± 33.92	0.001	
	Mangosteen obese	504.00 ± 14.85	0.006	0.179
	Curcumin obese	524.00 ± 21.63	0.001	0.813
	Synergistic obese	464.00 ± 38.19	0.001	0.023
Weight gain (g)	Control lean	152.00 ± 30.83		
	Mangosteen lean	210.80 ± 72.81		0.153
	Curcumin lean	207.20 ± 46.40		0.058
	Synergistic lean	207.00 ± 13.25		0.006
	Control obese	458.60 ± 35.66	0.001	
	Mangosteen obese	405.00 ± 16.67	0.003	0.016
	Curcumin obese	411.20 ± 21.05	0.001	0.034
	Synergistic obese	392.00 ± 38.63	0.001	0.022
BMI (g/cm^2^)	Control lean	0.55 ± 0.04		
	Mangosteen lean	0.61 ± 0.06		0.102
	Curcumin lean	0.65 ± 0.09		0.065
	Synergistic lean	0.57 ± 0.02		0.423
	Control obese	0.86 ± 0.09	0.001	
	Mangosteen obese	0.81 ± 0.06	0.001	0.360
	Curcumin obese	0.78 ± 0.03	0.030	0.105
	Synergistic obese	0.79 ± 0.07	0.001	0.259

^a^Significant difference between normal weight and obese rats in each group (control, mangosteen, curcumin, and synergistic).

^b^Significant difference between each group and respective normal weight or obese control groups.

### Effect of treatments on oxidative stress and antioxidant related variables

Levels of malondialdehyde (MDA) serve as a marker of lipid peroxidation and oxidative stress. Glutathione (GSH) levels, GST activity, and vitamin C levels also serve as markers of anti-oxidative capacity. Unexpectedly, obese controls did not show any significant increase in MDA levels or decrease in GSH levels, GST activity, or vitamin C levels (Table 2). While MPE did not affect these antioxidant markers in normal weight rats, it induced a significant increase of GSH in obese rats (123.86 ± 15.53 μg/ml vs 288.72 ± 121.37 μg/ml). There was also a significant increase in GST activity in rats treated with both mangosteen extract and curcumin (*p* ˂ 0.016), although independently these compounds did not achieve a significant effect. On the other hand, vita min C levels did not demonstrate any change either in HFD or in treated animals (Table 2).

**Table 2:** Independent and synergistic effects of curcumin and mangosteen extract on oxidative stress- and antioxidant-related markers in lean and HFD-induced obese rats.

Parameters	Groups	Mean ± SD	p-value^a^	p-value^b^
MDA (µg/ml)	Control lean	108.95 ± 12.54		
	Mangosteen lean	100.18 ± 0.98		0.193
	Curcumin lean	94.77 ± 8.12		0.067
	Synergistic lean	93.17 ± 11.36		0.071
	Control obese	94.97 ± 13.70	0.131	
	Mangosteen obese	81.21 ± 23.90	0.114	0.296
	Curcumin obese	94.73 ± 24.01	0.997	0.984
	Synergistic obese	101.98 ± 9.61	0.222	0.377
GSH (µg/ml)	Control lean	148.31 ± 36.42		
	Mangosteen lean	123.86 ± 15.53		0.205
	Curcumin lean	125.11 ± 16.23		0.230
	Synergistic lean	162.51 ± 47.20		0.609
	Control obese	168.50 ± 20.49	0.312	
	Mangosteen obese	288.72 ± 121.37	0.017	0.047
	Curcumin obese	140.94 ± 8.25	0.088	0.024
	Synergistic obese	152.84 ± 36.07	0.726	0.423
GST activity (µ/ml)	Control lean	1.35 ± 0.42		
	Mangosteen lean	1.73 ± 0.39		0.115
	Curcumin lean	1.23 ± 0.27		0.599
	Synergistic lean	0.68 ± 0.86		0.249
	Control obese	1.64 ± 1.46	0.600	
	Mangosteen obese	2.77 ± 2.33	0.599	0.346
	Curcumin obese	1.37 ± 0.23	0.293	0.465
	Synergistic obese	2.39 ± 0.53	0.016	0.117
Vit. C (µg/ml)	Control lean	0.04 ± 0.01		
	Mangosteen lean	0.05 ± 0.03		0.602
	Curcumin lean	0.07 ± 0.09		0.675
	Synergistic lean	0.03 ± 0.01		0.094
	Control obese	0.03 ± 0.00	0.075	
	Mangosteen obese	0.02 ± 0.01	0.016	0.115
	Curcumin obese	0.06 ± 0.08	0.141	0.462
	Synergistic obese	0.03 ± 0.01	0.402	0.600

^a^Significant difference between normal weight and obese rats in each group (control, mangosteen, curcumin, and synergistic).

^b^Significant difference between each group and respective normal weight or obese control groups.

### Effect of treatments on absolute and relative levels of dyslipidemia related variables

Dyslipidemia was measured by blood cholesterol, TAG, LDL-C, and HDL-C levels. Obese rats displayed significantly higher cholesterol, TAG, and LDL-C levels, and non-significantly lower HDL-C levels (Table 3). While MPE was ineffective in reducing dyslipidemia-related markers (total and LDL-C), it did induce a significant increase in HDL-C, an anti-atherogenic marker (*p* ˂ 0.026).

**Table 3:** Independent and synergistic effects of curcumin and mangosteen extract on the lipid profiles of lean and HFD-induced obese rats.

Parameters	Groups	Mean ± SD	p-value^a^	p-value^b^
CHOL (mg/dl)	Control lean	74.26 ± 6.97		
	Mangosteen lean	105.51 ± 14.21		0.002
	Curcumin lean	104.07 ± 9.84		0.001
	Synergistic lean	88.87 ± 2.93		0.003
	Control obese	148.00 ± 48.24	0.026	
	Mangosteen obese	182.22 ± 9.31	0.001	0.189
	Curcumin obese	123.41 ± 34.60	0.287	0.381
	Synergistic obese	145.97 ± 21.93	0.004	0.934
HDL-C (mg/dl)	Control lean	54.95 ± 8.71		
	Mangosteen lean	72.29 ± 17.96		0.088
	Curcumin lean	56.18 ± 11.46		0.853
	Synergistic lean	68.57 ± 18.27		0.171
	Control obese	51.45 ± 17.76	0.702	
	Mangosteen obese	55.08 ± 8.81	0.026	0.693
	Curcumin obese	50.16 ± 12.30	0.446	0.897
	Synergistic obese	50.94 ± 13.88	0.124	0.961
LDL-C (mg/dl)	Control lean	11.12 ± 3.89		
	Mangosteen lean	31.88 ± 12.21		0.016
	Curcumin lean	30.30 ± 10.07		0.009
	Synergistic lean	13.19 ± 3.25		0.463
	Control obese	65.05 ± 43.59	0.075	
	Mangosteen obese	95.94 ± 8.81	0.009	0.173
	Curcumin obese	49.35 ± 16.88	0.076	0.602
	Synergistic obese	58.45 ± 15.91	0.009	0.754
TAG (mg/dl)	Control lean	182.29 ± 31.89		
	Mangosteen lean	194.60 ± 24.94		0.516
	Curcumin lean	196.49 ± 18.04		0.411
	Synergistic lean	215.54 ± 6.71		0.052
	Control obese	223.07 ± 14.77	0.032	
	Mangosteen obese	222.53 ± 3.91	0.038	0.941
	Curcumin obese	197.22 ± 89.32	0.987	0.556
	Synergistic obese	250.27 ± 44.94	0.126	0.234

^a^Significant difference between normal weight and obese rats in each group (control, mangosteen, curcumin, and synergistic).

^b^Significant difference between each group and respective normal weight or obese control groups.

Obese rats also had higher total cholesterol/HDL-C and HDL-C/LDL-C values, clinically used measures that indicate a risk factor for cardiovascular disease. While normal weight rats recorded ratios of 1.52 ± 0.28 and 4.62 ± 1.21, respectively, obese rats recorded much higher total cholesterol/HDL-C (3.02 ± 0.78) concomitant with much lower HDL-C/LDL-C (1.15 ± 0.73) ratios (Table 4). Serum glucose was also measured in the eight experimental groups. While HFD-induced obesity did not lead to elevation of blood glucose, MPE was effective in reducing glucose levels in both normal weight and obese animals reporting values of 154.43 ± 34.27 and 169.67 ± 36.21 respectively which is much lower than control untreated lean and obese animals. The synergistic treatment domonstrate more potency in reducing blood glucose levels in HFT-induced obese rats recording value of 155.44 ± 74.38 (Table 4).

**Table 4:** Independent and synergistic effects of curcumin and mangosteen extract on cardiovascular risk factors in lean and HFD-induced obese rats.

Parameters	Groups	Mean ± SD	p-value^a^	p-value^b^
CHOL/HDL-C	Control lean	1.52 ± 0.28		
	Mangosteen lean	1.49 ± 0.24		0.877
	Curcumin lean	1.73 ± 0.40		0.352
	Synergistic lean	1.36 ± 0.32		0.416
	Control obese	3.02 ± 0.78	0.010	
	Mangosteen obese	2.97 ± 0.85	0.005	0.934
	Curcumin obese	2.67 ± 1.31	0.165	0.628
	Synergistic obese	2.78 ± 0.67	0.003	0.620
HDL-C/LDL-C	Control lean	4.61 ± 1.21		
	Mangosteen lean	2.54 ± 1.02		0.016
	Curcumin lean	1.95 ± 0.40		0.009
	Synergistic lean	5.35 ± 1.43		0.465
	Control obese	1.15 ± 0.73	0.009	
	Mangosteen obese	0.57 ± 0.07	0.009	0.346
	Curcumin obese	1.21 ± 0.79	0.117	0.917
	Synergistic obese	0.95 ± 0.51	0.009	1.000
GLU (mg/dl)	Control lean	232.55 ± 74.67		
	Mangosteen lean	154.43 ± 34.27		0.076
	Curcumin lean	121.12 ± 8.90		0.009
	Synergistic lean	162.08 ± 39.89		0.117
	Control obese	206.94 ± 100.22	0.347	
	Mangosteen obese	169.67 ± 36.21	0.346	0.917
	Curcumin obese	174.43 ± 56.07	0.076	0.754
	Synergistic obese	155.44 ± 74.38	0.917	0.754

^a^Significant difference between normal weight and obese rats in each group (control, mangosteen, curcumin, and synergistic).

^b^Significant difference between each group and respective normal weight or obese control groups.

Correlations between all measured markers were also calculated. Negative correlations were observed between BMI, antioxidant status (GSH levels), and HDL-cholesterol (Table 5). BMI was also positively correlated with dyslipidemia-related markers CHOL, LDL, TAG, and CHOL/HDL-C with strong P values < 0.01 or 0.05.

**Table 5:** Pearson’s correlation coefficients between measured variables.

Parameters	R (correlation Coefficient)	p-value	Correlation
BMI with CHOL	0.601**	0.000	P^a^
BMI with HDL-C	− 0.381*	0.015	N^b^
BMI with GSH	− 0.298*	0.011	N^b^
BMI with CHOL/HDL-C	0.645**	0.000	P^a^
CHOL with TRIG	0.507**	0.001	P^a^
TRIG with CHOL/HDL-C	0.384*	0.014	P^a^
LDL-C with BMI	0.634**	0.000	P^a^
LDL-C with GSH	0.343*	0.030	N^a^
LDL-C with CHOL	0.955**	0.000	P^a^
LDL-C with TRIG	0.451**	0.003	P^a^
LDL-C with HDL-C/LDL-C	− 0.927**	0.000	N^b^

**p < 0.01.

*p < 0.05.

^a^Positive correlation.

^b^Negative correlation.

ROC and AUC were calculated to measure specificity and sensitivity in the four obese groups. Among the measured variables, BMI was the most efficient variable either to predict obesity in HFD-fed mice or the therapeutic potency of curcumin and mangosteen (Table 6). With exception of glucose most of the measured variables recorded excellent ROCAUCs range between 0.8 and 1.0 with satisfactory specificity and sensitivity.

**Table 6:** ROC of all parameters for obese rats.

Parameters	Groups	AUC	Cut-off value	Sensitivity %	Specificity %	P value	95% CI
BMI	Control obese	1.000	0.705	100.0	100.0	0.009	1.000–1.000
	Mangosteen obese	1.000	0.695	100.0	100.0	0.009	1.000–1.000
	Curcuma obese	1.000	0.755	100.0	100.0	0.009	1.000–1.000
	Synergistc obese	1.000	0.660	100.0	100.0	0.009	1.000–1.000
MDA	Control obese	0.800	102.640	80.0	80.0	0.117	0.494–1.106
	Mangosteen obese	0.800	98.195	80.0	100.0	0.117	0.449–1.151
	Curcuma obese	0.520	110.025	40.0	100.0	0.917	0.110–0.930
	Synergistc obese	0.760	90.030	100.0	60.0	0.175	0.436–1.084
GSH	Control obese	0.680	148.060	100.0	60.0	0.347	0.301–1.059
	Mangosteen obese	1.000	149.995	100.0	100.0	0.009	1.000–1.000
	Curcuma obese	0.840	135.745	80.0	80.0	0.076	0.580–1.100
	Synergistc obese	0.580	162.795	80.0	60.0	0.676	0.195–0.965
GST	Control obese	0.600	0.911	40.0	100.0	0.602	0.219–0.981
	Mangosteen obese	0.600	2.417	60.0	100.0	0.602	0.171–1.029
	Curcuma obese	0.700	1.228	80.0	60.0	0.296	0.358–1.042
	Synergistc obese	0.960	1.432	100.0	80.0	0.016	0.843–1.077
Vit. C	Control obese	0.840	0.028	80.0	80.0	0.076	0.580–1.100
	Mangosteen obese	0.960	0.035	100.0	80.0	0.016	0.843–1.077
	Curcuma obese	0.780	0.024	60.0	100.0	0.144	0.467–1.093
	Synergistc obese	0.660	0.032	60.0	80.0	0.403	0.304–1.016
CHOL	Control obese	1.000	85.105	100.0	100.0	0.009	1.000–1.000
	Mangosteen obese	1.000	146.145	100.0	100.0	0.009	1.000–1.000
	Curcuma obese	0.640	126.625	60.0	100.0	0.465	0.238–1.042
	Synergistc obese	1.000	109.055	100.0	100.0	0.009	1.000–1.000
HDL-C	Control obese	0.560	58.545	60.0	80.0	0.754	0.168–0.952
	Mangosteen obese	0.920	56.185	80.0	100.0	0.028	0.738–1.102
	Curcuma obese	0.640	60.955	100.0	40.0	0.465	0.277–1.003
	Synergistc obese	0.800	59.835	80.0	80.0	0.117	0.494–1.106
LDL-C	Control obese	0.840	29.425	80.0	100.0	0.076	0.544–1.136
	Mangosteen obese	1.000	65.210	100.0	100.0	0.009	1.000–1.000
	Curcuma obese	0.840	44.870	80.0	100.0	0.076	0.544–1.136
	Synergistc obese	1.000	27.970	100.0	100.0	0.009	1.000–1.000
TRIG	Control obese	1.000	204.290	100.0	100.0	0.009	1.000–1.000
	Mangosteen obese	0.960	211.535	100.0	80.0	0.016	0.843–1.077
	Curcuma obese	0.600	226.895	60.0	100.0	0.602	0.171–1.029
	Synergistc obese	0.800	242.755	80.0	100.0	0.117	0.449–1.151
CHOL/HDL-C	Control obese	1.000	1.950	100.0	100.0	0.009	1.000–1.000
	Mangosteen obese	1.000	1.800	100.0	100.0	0.009	1.000–1.000
	Curcuma obese	0.800	1.970	80.0	80.0	0.117	0.494–1.106
	Synergistc obese	1.000	1.790	100.0	100.0	0.009	1.000–1.000
HDL-C /LDL-C	Control obese	1.000	2.470	100.0	100.0	0.009	1.000–1.000
	Mangosteen obese	1.000	1.065	100.0	100.0	0.009	1.000–1.000
	Curcuma obese	0.800	1.305	80.0	100.0	0.117	0.449–1.151
	Synergistc obese	1.000	2.605	100.0	100.0	0.009	1.000–1.000
GLU	Control obese	0.680	152.088	60.0	100.0	0.347	0.301–1.059
	Mangosteen obese	0.680	145.168	80.0	60.0	0.347	0.328–1.032
	Curcuma obese	0.840	141.216	60.0	100.0	0.076	0.580–1.100
	Synergistc obese	0.520	139.069	80.0	40.0	0.917	0.135–0.905

Multiple regression analysis was also performed using GSH levels as the dependent variable. Interestingly, we observed that MDA as measure of oxidative stress together with dyslipidemia-related independent variables greatly contributed to GSH depletion (Table 7).

**Table 7:** Multiple regression analysis using GSH levels as the dependent variable.

Predictor variable	Coefficient	SE	p-value	Adjusted R^2^	95% CI	
					Lower	Upper
LDL-C	1.196	0.285	0.000	0.298	0.618	1.773
LDL-C	2.355	0.391	0.983	0.482	1.563	3.146
HDL-C/LDL-C	25.584	6.716	0.000		11.976	39.191
LDL-C	2.231	0.350	0.000	0.589	1.521	2.941
HDL-C/LDL-C	26.558	5.990	0.000		14.409	38.707
MDA	− 1.509	0.463	0.002		− 2.448	− 0.570

## Discussion

A diet high in fat leads to obesity in both humans and animals^19,20^. In both rats and mice, a positive relationship has been demonstrated between the levels of fat in the diet and body weight or fat gain; also, rats that consumed diets containing high quantities of fat gained weight faster than those on diets containing minimal fat^21–25^.

The recorded increase of BMI in obese rats is in agreement with previous studies^26^, in which they reported that BMI in obese rats is usually higher than 0.75 gm/cm^2^. The final weight and weight gain in obese rats were also coupled with high BMI, in agreement with the findings of Picklo et al.^27^, who showed the obesogenic effect of a saturated lipid diet in animal models. Unexpectedly, while both MPE and curcumin were effective in lowering the BMI of obese animals, both also induced weight gain in lean animals (Table 1 and Fig. 1). This is similar to the findings of Husen et al.^28^, who reported an increase in body weight following mangosteen extract and curcumin after the decrease in body weight induced by STZ treatment.

The elevated levels of ROS after high caloric intake or inflammation can later result in increase of BMI or incidence of obesity. Adipose tissue can undergo pathological alterations induced by inflammation or oxidative stress, which in turn enhances the secretion of adipokines and affects the peripheral tissues that produce ROS, further promoting oxidative stress and the inflammatory response^29,30^. The concentration of the serum MDA can be used as an indicator of oxidative stress. MDA is one of the final products of the peroxidation of polyunsaturated fatty acids (PUFA). The concentration of MDA can be used as an indicator of cell or tissue damage due to the increase in lipid peroxidation. The unexpected non-significant increase of MDA and decrease in GSH levels, GST activity, and vitamin C levels in HFD-induced obese rats might be attributed to the addition of coconut oil to the high saturated fat diet given in our paradigm as an inducer of obesity. Multiple studies demonstrate the anti-oxidative effects of virgin coconut oil, supporting this hypothesis^31,32^. Table 2 also shows the independent and synergistic effects of MPE and curcumin on oxidative stress-related variables. While MPE did not demonstrate anti-oxidative effects in normal weight rats, it did induce a significant increase in GSH levels in obese rats. GST functions as antioxidant, and enzymes can catalyze the detoxification of xenobiotics via conjugation with GSH.

The diet-induced obesity animal model is one of the most common and reliable models used in obesity studies due to its similarity in modeling the most common route of obesity in humans, as well as related metabolic effects. As shown previously, this HFD models obesity via increased food intake, body weight gain, body fat accumulation, BMI increase, defects in antioxidant levels, and disruption in the lipid profile^19,25^.

While LDL-C is responsible for the delivery of cholesterol to peripheral tissues, HDL-C mediates the inverse process of cholesterol transport from peripheral tissues^33^. The non-significant decrease of HDL-C in obese rats reported in the present study may be related to the anti-oxidative effect of coconut oil, a component of the HFD used here^31,32^.

In general, ingestion of coconut oil can increase HDL-C^34^. It has been proposed that lauric acid, the main constituent of coconut oil, is the cornerstone of this pathway. Lauric acid accounts for 50% of the content of coconut oil. Although lauric acid is considered a medium-chain fatty acid (MCFA), 70% of lauric acid is transported as a long-chain fatty acid (LCFA), while the other 30% remains as a MCFA. Thus, there are two ways of transporting lauric acid in the body. When lauric acid reaches the liver, it serves as a substrate in the production of apoA1 and apoB, further contributing to the formation of both HDL-C and LDL-C^35^.

Studies show that in contrast, to LDL-C, HDL-C may play an anti-atherogenic and anti-thrombotic role by protecting LDL-C particles against lipid peroxidation and reducing the deleterious effects of oxidized LDL-C^35^. Based on this report, the non-significant decrease of HDL-C in obese rats may be related to the presence of coconut oil in the HFD^36^.

Moreover, our data is consistent with those of Wihastuti et al.^37^, who showed that 400 mg/kg body weight MPE affected LDL-C but no other lipid marker, and significantly reduced H_2_O_2_ levels and NF-κB expression. At concentrations of 800 mg/kg body weight, this extract was most effective in improving the lipid profile; this suggests that although in the present study MPE did induce a hypo-lipidemic effect in obese rats, the anti-atherogenic effects likely occurred via its anti-oxidative and anti-inflammatory effects^37^.

This contradiction may also be attributed to the fact the amount of total xanthone in the MPE is strongly affected by the extraction capacity of the solvent to recover different phenolic constituents from various fruit origins, as well as the methods of transportation and storage^38–40^. Aisha et al.^41^ have reported that toluene is the most efficient extraction solvent for MPE compared to 75% ethanol and methanol^42^.

Obesity is a risk factor for the development of cardiovascular disease, diabetes mellitus, hyperlipidemia, and arteriosclerosis^43^. To treat and prevent obesity and obesity-related complications, an increasing number of people use hypoglycemia-inducing or weight-loss drugs. However, the long-term use of these drugs can damage the liver and kidney. Unsurprisingly, finding safe and effective weight-loss- and hypoglycemia-inducing agents is becoming increasingly urgent.

Table 4 reveals the level of serum glucose in the studied rats. While obesity itself did not induce elevation of blood glucose, MPE was effective in reducing glucose levels in both normal weight and obese animals. This is consistent with the previous work of Taher et al.^44^, which demonstrated that orally administered MPE at various doses demonstrated a hypoglycemic effect in streptozotocin (STZ)-induced diabetic rats and normoglycemic rats. Moreover, curcumin demonstrates hypoglycemic effects in both obese and normal weight rats; in obese rats, the synergistic effects of both of these extracts was much higher than each extract independently. This is also supported by Rivera-Mancía et al.^45^ and Sohaei et al.^46^, who showed the hypoglycemic effects of curcumin. As previously described, the use of curcumin in vitro and in animal models of diabetes revealed a variety of potential mechanisms of action to treat diabetes mellitus; however, clinical trials in humans have thus far been inconsistent with these findings.

Pearson’s correlation coefficient (PCC) is a statistical metric that measures the strength and direction of a linear relationship between two or more random variables^47^. Table 5 indicates the correlations between all variables measured in this study. It can be seen that obesity (BMI), antioxidant status (GSH levels), and dyslipidemia-related markers (CHOL, TAG, CHOL/HDL-C) were negatively or positively correlated in a manner that demonstrates the negative impact of oxidative stress and dyslipidemia on obesity.

Abruzzo et al.^48^ highlighted the advantage of using ROC curves as an outstanding statistical tool for the identification of biomarkers that are sufficiently sensitive and specific for the early diagnosis of obesity. Although its utility in prediction, risk valuation, and assessment of therapeutic interventions still requires further validation, ROC curves emphasize the most significant statistical differences between patients and controls and even animal models of diseases^49^. The AUC provides a useful measure to evaluate the predictive value of biomarkers. While an AUC value near 1 designates an excellent predictive marker, a curve that lies adjacent to the diagonal (AUC = 0.5) indicates no diagnostic usefulness. AUC values close to 1.00 are always accompanied by satisfactory values of specificity and sensitivity^50^.

Table 6 demonstrates the ROC curves with AUC, specificity, and sensitivity in obese rats. Among the measured variables, BMI showed excellent predictive value as a marker of obesity, with AUC between 0.8 and 1 with satisfactory specificity and sensitivity. The other measured variables demonstrate relatively less predictive ability with any specificity and sensitivity.

## Conclusion

The present study ascertained the efficiency of a HFD in inducing obesity in rats, and the effectiveness of MPE and curcumin independently or synergistically in treating obesity-induced atherogenesis.

## Methods

### Preparation of mangosteen extract

Mangosteen fruits were obtained from a local market in Riyadh, and authenticated by a taxonomist from the Department of Botany, College of Science, King Saud University, Riyadh. 500 g of the dried pericarp of mangosteen was soaked in 1.5 L of 99.7% methanol at room temperature for 72 h. Extracts were filtered through Whatman No. 1 filter paper, and solvent was evaporated using a rotary evaporator at 60 °C. The resulting viscous concentrate was freeze-dried to ensure complete removal of solvent, and stored at − 20 °C for further use. GC–MS analysis for *Garcinia mangostina* was done and show 99–95% similarity in the database for fatty acids. Major components of the extract are listed in Table S1 shown below.

### Preparation of curcumin

Fresh curcumin was obtained from a local market in Riyadh, and dried after proper cleaning using a drying oven with an air fan at 50 °C for 3–5 h. Curcumin was then ground and sieved, and the remaining soft powder was stored at − 80 °C until further use.

### Animals

All experimental procedures were carried out in Experimental Surgery and Animal Laboratory Prince Naif Health Research Center (PNHRC) after being approved by the Ethical Committee of Scientific Research at KSU. All animals hosted in in polypropylene cages in an environmentally controlled clean air room, with a temperature of (25 °C ± 1 °C) a 12 h light/12 h dark cycle and a relative humidity of 50 ± 5%.

Forty male albino Wistar rats, weighing 100 ± 20 g of four weeks age, The animals had free access food (standard laboratory animal feed pellets) and water for 4 groups normal weight and HFD for another four groups to induce obesity. The diet was prepared in collaboration with (PNHRC), Medicine college in King Khalid Hospital (KKH), in (KSU), HFD (45% kcal from fat) was prepared by making the composition of the British company. Test Diet replacing the pig fat in the composition of the company with 50% hydrogenated fats (Butter oil, Palm oil) and 50% coconut oil due as pork fat is forbidden according to the Saudi Food and Drug Regulations SFD (Diet formulation is shown in Table S2). All experiments were performed in accordance with the guidelines of the National Institutes of Health for the Care and Use of Laboratory Animals and approved by the Animal Ethics Committee of King Saud University (reference no: KSU-SE-18-17).

### Animal groups and diets

Animals were divided into eight groups of 5 animals each. Group 1 serves as the control group, as animals were fed a normal diet. Group 2 received a normal diet and 400 mg/kg body weight/day water extract of mangosteen pericarp for 6 weeks. Group 3 rats received a normal diet and curcumin at 80 mg/kg body weight/day for 6 weeks. Group 4 received a normal diet and both MPE (400 mg/kg body weight/day) and curcumin (80 mg/kg body weight/day) for 6 weeks. Group 5 was fed with a high-fat diet (HFD) for 4 weeks. Group 6 rats were fed a HFD for 4 weeks followed by 400 mg/kg body weight/day water extract of mangosteen pericarp for 6 weeks^51^. Group 7 received HFD for 4 weeks, followed by 80 mg/kg body weight/day curcumin for 6 weeks^52^. Group 8 animals received a HFD for 4 weeks, followed by both MPE (400 mg/kg body weight/day) and curcumin (80 mg/kg body weight/day) for 6 weeks. At the end of the experiment, animals were anesthetized with 5.0% of sevoflurane and 100% oxygen, the flow rate of sevoflurane was determined as the following formula: flow rate (ml/min) = 0.5 × body weight (g). Blood was collected from the eye and heart using hematocrit capillaries (75 mm/75 μl). Our study was carried out in compliance with the ARRIVE guidelines.

### Collection of serum

Serum was collected from blood samples by centrifugation for 15 min at 4000 rpm. Serum was further utilized in the analysis of the following biochemical parameters.

### Levels of serum lipid fractions

Cholesterol, triglyceride, HDL, and LDL levels were quantitatively determined using commercial kits from United Diagnostics Industry Company (UDIC), Riyadh, Saudi Arabia.

### Levels of serum glucose

Quantitative determination of glucose levels was made via the use of Trinder glucose oxidase I activity test using a kit from, a product of United Diagnostics Industry Company (UDIC), Riyadh, Saudi Arabia.

### Level of serum oxidative stress markers

Lipid oxidation was estimated according to the method reported by Begonaruizlarrea et al.^53^. In this method, lipid peroxidation was determined by measuring the thiobarbituric acid reactive sbstances (TBARS), mainly malondialdehyde (MDA).

Vitamin C was estimated using the method described by Jagota and Dani^54^. In this method, ascorbic acid was reacted Folin phenol reagent to develop a color which is directly proportional to vitamin C concentration. The absorption maximum of the color developed by the interaction of ascorbic acid with Folin reagent is 760 nm. Glutathione levels were evaluated per the method described by Beutler et al.^55^. The method based on the development of a relatively stable yellow color when 5, 5-dithiobis-2-nitrobenzoic acid (DTNB) was added to sulphahydryl compounds. Glutathione S-transferase (GST) activity was determined by method reported by Habig and Pabst [^56^]. The reaction is measured by observing the conjugation of 1-chloro, 2, 4-dinitrobenzene (CDNB) with reduced (GSH). This was done by watching an increase in absorbance at 340 nm.

### Statistical analysis

Data are expressed as means ± standard deviation (SD). To compare results between groups, one-way analysis of variance (ANOVA) tests were used. Significance was assigned at the level of *p* < 0.05. The receiver operating characteristics curve (ROC) was analyzed, and the area under the curve (AUC), cutoff values, and degrees of sensitivity and specificity were calculated.

## Supplementary Information

Supplementary Information
