# Supplementary material for: Garcinia mangostana extract and curcumin ameliorate oxidative stress, dyslipidemia, and hyperglycemia in high fat diet-induced obese Wistar albino rats
Source: Sci Rep. 2021 Mar 31;11:7278. doi: 10.1038/s41598-021-86545-z (PMC8012579; doi:10.1038/s41598-021-86545-z)
Supplement: Supplementary file 1 — Supplementary Information [file 41598_2021_86545_MOESM1_ESM.docx]

Table S1. Composition analysis using GC-MS for *Garcinia mangostina* shown 99-95% similarity in the database for fatty acids.

| **Component** | **Percent Area (%)** | **Retention**  **Time** | **Peak** |
| --- | --- | --- | --- |
| p-Dioxane-2,3-diol 2(R),3(S)-1,2,3,4-Butanetetrol Glycerin | 1.64 | 3.489 | 1 |
| 2-Butanone, 4-hydroxy-2-Propanol, 1-amino-Oxirane | 1.52 | 4.287 | 2 |
| 4-hydroxy-3-methyl-2-Butanone | 8.57 | 4.330 | 3 |
| 2-Furanmethanol | 1.02 | 5.672 | 4 |
| 1H-Imidazole, (6)-thiophene-3-sulfonyl)-piperidin | 0.60 | 6.496 | 5 |
| 2,3-dihydro-2-hydroxy- 1-Methyl-2-piperidinemethanol 4-Pyranone. | 1.34 | 6.640 | 6 |
| 5-Methyloxazolidine, Ethanone, 1-(3-ethyloxiranyl)-3-Buten-2-ol | 0.52 | 12.910 | 7 |
| .alpha.-Cubebene | 3.55 | 16.214 | 8 |
| (-)-.alpha.-Panasinsen | 0.87 | 18.355 | 9 |
| 3-Hydroxy-1-propenyl)-2-methoxyphenol 3,7-Benzofurandiol, 2-dimethyl-Pyrazine, | 0.45 | 20.997 | 10 |
| Hexadecanoic acid, methyl ester | 1.08 | 22.883 | 11 |
| n-Hexadecanoic acid | 2.37 | 23.240 | 12 |
| Cyclononasiloxane, octadecamethyl- Cyclodecasiloxane, | 0.31 | 23.427 | 13 |
| 3,5-di-tert-Butyl-4-hydroxyphenylpropionic acid | 0.62 | 23.614 | 14 |
| 9,12-Octadecadienoic acid (Z,Z)-, methyl ester | 0.60 | 24.531 | 15 |
| 9-Octadecenoic acid (Z)-, methyl ester | 0.92 | 24.574 | 16 |
| Octadecanoic acid, methyl ester | 0.85 | 24.795 | 17 |
| cis-Vaccenic acid | 3.77 | 24.931 | 18 |
| Octadecanoic acid | 1.75 | 25.118 | 19 |
| Pentasiloxane | 0.44 | 28.439 | 20 |
| Germacyclopentane, | 5.72 | 33.010 | 21 |
| Silicic acid, diethyl bis(trimethylsilyl) ester | 2.82 | 34.836 | 22 |

Table S2. Diet Formulation:

| **Diet Type** | **Standard Diet** | **High Fat Diet**  **(45.6% kcal from fat)** |
| --- | --- | --- |
| Ingredients | g/kg | g/kg |
| Casein | 140 | 200 |
| Corn starch | 465.56 | 317.86 |
| Sucrose | 100 | 80 |
| Maltodextrin | 145 | 23 |
| Cellulose | 50 | 65 |
| Soy oil | 50 | 33 |
| Calcium phosphate | - | 17 |
| Minerals Mix. | 35 | 13 |
| Vitamins Mix. | 10 | 10 |
| L-Cystine | 1.8 | 4 |
| Choline | 2.5 | 3 |
| Calcium carbonate | - | 3 |
| Fats source ( Butter oil, Coconut Oil, Palm oil) | - | 230 |
| Cholesterol | - | 1 |
| tBHQ, tert-Butylhydroquinone | 0.14 | 0.14 |
| Total | 1KG | 1KG |
